# Identifying Atrial Fibrillation Mechanisms for Personalized Medicine

**DOI:** 10.3390/jcm10235679

**Published:** 2021-12-01

**Authors:** Brototo Deb, Prasanth Ganesan, Ruibin Feng, Sanjiv M. Narayan

**Affiliations:** Division of Cardiovascular Medicine, Department of Medicine, Stanford University, Mailcode 5687, 453 Quarry Road, Palo Alto, CA 94304, USA; bdeb7@stanford.edu (B.D.); prash030@stanford.edu (P.G.); ruibin@stanford.edu (R.F.)

**Keywords:** atrial fibrillation, precision medicine, personalized therapy, machine learning, artificial intelligence, pathophysiology

## Abstract

Atrial fibrillation (AF) is a major cause of heart failure and stroke. The early maintenance of sinus rhythm has been shown to reduce major cardiovascular endpoints, yet is difficult to achieve. For instance, it is unclear how discoveries at the genetic and cellular level can be used to tailor pharmacotherapy. For non-pharmacologic therapy, pulmonary vein isolation (PVI) remains the cornerstone of rhythm control, yet has suboptimal success. Improving these therapies will likely require a multifaceted approach that personalizes therapy based on mechanisms measured in individuals across biological scales. We review AF mechanisms from cell-to-organ-to-patient from this perspective of personalized medicine, linking them to potential clinical indices and biomarkers, and discuss how these data could influence therapy. We conclude by describing approaches to improve ablation, including the emergence of several mapping systems that are in use today.

## 1. Introduction

Atrial fibrillation (AF) is the most prevalent cardiac arrhythmia, affecting 1–2% of the US population [1]. The worldwide burden of AF is estimated at 30 million and is increasing in parallel with the aging of the population and the epidemics of metabolic syndrome, hypertension, and other various and less clear [2] risk factors. About a third of cases are asymptomatic (silent AF), based on implanted loop recorder studies [3]. 

AF is defined by its typical electrocardiogram (ECG) appearance that lacks distinct atrial activations (P waves) and shows rapid, irregular atrial waveforms (f-waves) with irregularly irregular QRS complexes [4]. However, this definition embodies diverse phenotypes that include, at a minimum, transient self-limited episodes of AF after a stressor (such as thyrotoxicosis or surgery), intermittent self-limiting episodes with no clear precipitant over a prolonged timeframe, or AF that progresses inexorably over time towards becoming continuous in otherwise healthy individuals, and AF in tandem with sleep apnea, heart failure, or other comorbidities. Thus, there is an urgent need to develop a framework that separates these and other AF presentations by their pathophysiology to personalize therapy for each patient. Recent studies applying artificial intelligence (AI) to the ECG and other data types offer such a foundation for computational phenotypes that could guide therapy [5,6].

**Figure 1:**
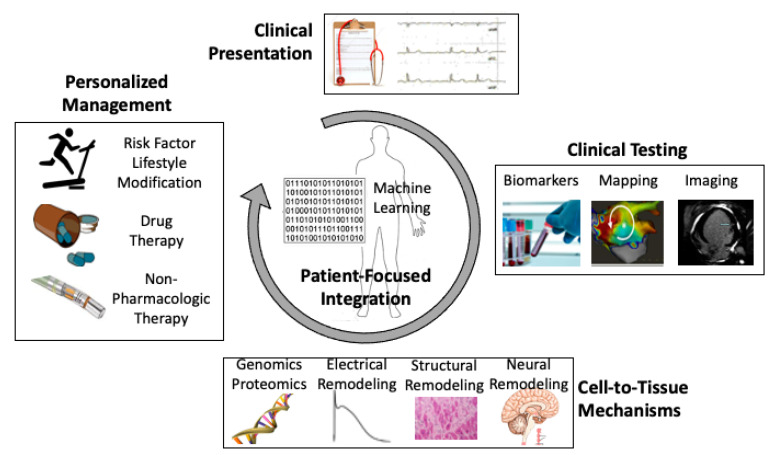
Patient-focused mechanistic integration to guide personalized atrial fibrillation (AF) management. Clinical presentation alone is suboptimal to guide care, but provides some clues to mechanistic subtypes. Phenotypes can be refined by clinical testing and invasive studies. Ideally, precision phenotypes that integrate mechanisms across multiple biological scales, including biomarkers of omic risk, electrical and structural remodeling, and continuous data streams from wearables, may enable personalized AF therapy. This may be facilitated by tools such as machine learning.

In this review, we attempt to synthesize AF mechanisms from the perspective of personalized medicine, covering bench to bedside discoveries at cell, organ, and patient levels [7,8]. The ultimate synthesis of the mechanisms to guide patient care will require clinical integration, potentially assisted by decision support systems such as machine learning models (Figure 1).

## 2. Risk Factors Provide Mechanistic Clues

AF is typically subdivided into paroxysmal and non-paroxysmal (including persistent, long-standing persistent, and permanent AF). Paroxysmal AF (PAF) is defined as self-terminating episodes lasting <7 days, persistent AF (PsAF) as episodes that last between 7 days to 1 year, and long-standing persistent AF as continuous AF lasting longer than one year [9]. While clinically useful, patients grouped by these detected AF episode durations overlap considerably in their actual AF burdens on implanted monitors [10] and their response to drug or ablation therapy [11,12,13,14,15]. Thus, these AF definitions only modestly separate the mechanisms. Non-modifiable risk factors for AF include age, sex (AF is more common in males), and genetics [16]. Potentially modifiable risk factors include metabolic disorders and obesity [17], obstructive sleep apnea [18], alcohol use [19], vigorous endurance exercise [20], sleep deprivation [21], and risk factors from the Framingham Heart Study, namely, hypertension, congestive HF, coronary artery disease, valvular heart disease, and diabetes mellitus [22,23]. 

While the existence of these comorbidities influences the risk for stroke and the need for anticoagulation, they do not substantially alter AF management. Clinical scores such as CHA_2_DS_2_-VASc and HATCH only modestly predict incident AF [24,25,26,27], outcomes from ablation [28,29] and, in fact, even risk for stroke [30]. Weight loss as a lifestyle intervention can reduce the symptoms and severity of AF [31]; however, this is less clear in patients with diabetes mellitus [32] or those with paroxysmal AF undergoing ablation [33].

## 3. Pathophysiology of AF at the Genetic Level

It is well established that individuals with familial AF and a first-degree relative with AF have an increased risk for incident AF [16]. This risk likely comes from two mechanisms. First, rare monogenic mutations in channels and gap junction proteins with large effect sizes occur in families with AF [34] and are more common in early-onset AF [35]. This includes germline mutations that pass to descendants as well as somatic mutations. When identified, such features motivate family screening to identify early-onset AF [35]. Second, common variations in a network of over 100 genes now identified from genome-wide association studies (GWAS) confer a smaller, yet additive, risk for AF [36]. However, these currently known genetic variants do not readily separate AF phenotypes nor explain the success from therapy [37]. It is hoped that a wider application of next generation sequencing, enhanced AF monitoring using mHealth technology, mechanistic studies focusing on gene networks, and machine learning to integrate multiomics data may, in the near term, reveal genomically informed phenotypes that guide patient management [38].

## 4. Pathophysiology for AF at the Cellular Level

**Figure 2:**
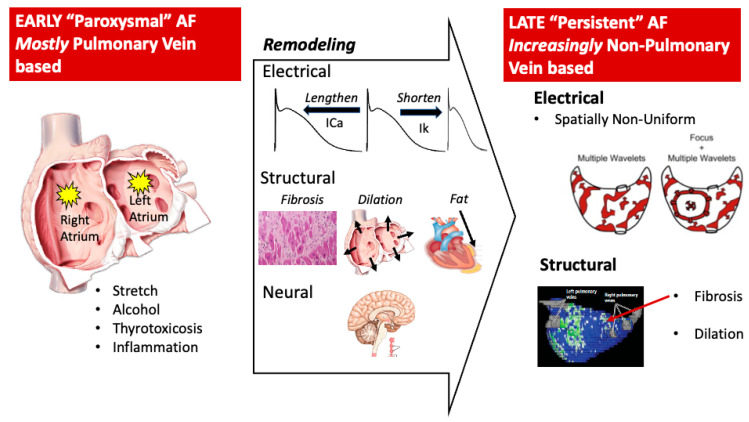
Progression of atrial fibrillation from a pulmonary vein–focused disease to a non-pulmonary vein–focused disease. Early AF is initiated by triggers near the pulmonary veins (PVs), that are exacerbated by stretch, inflammation, and other factors. The remodeling of electrical, structural, and neural elements can increasingly be measured in patients. Late AF is characterized by substrates that maintain AF, which are often located outside the pulmonary veins, and likely involve an interplay between electrical and structural components. Bottom right figure is reproduced with permission from Marrouche et al. [39].

Several cellular mechanisms for AF have been described at the electrical, structural, and autonomic levels, although few biomarkers have been defined to identify which is operative in any one patient. In general, AF is triggered and sustained due to several forms of remodeling (Figure 2).

### 4.1. Electrical Modeling

Electrical remodeling in AF patients is indicated by altered atrial refractory periods due to changes in Ca^2+^ currents and outward K^+^ currents [40,41], and conduction slowing from an altered expression and the localization of connexins between myocytes [42]. These factors interact with structural remodeling (see below), ischemia, stretch, and autonomic stimuli to facilitate ectopic triggers from the PVs and other regions, and may maintain AF by promoting re-entry or focal beats. These mechanisms explain the success of pharmacotherapy in some patients to block Na^+^ (class I agents), K^+^ (class III agents), or Ca^2+^ mechanisms (class IV agents) in AF. It is unclear, however, how best to use the knowledge regarding these mechanisms to guide optimal pharmacotherapy in any one individual.

Abnormal calcium signaling is a well-established mechanism in AF. Abnormalities in subcellular Ca^2+^-dependent signaling and in Ca^2+^/calmodulin-dependent protein kinase II (CaMKII) activity can cause triggered ectopy [43] and affect the calcium (ICa) current, which alters atrial refractory periods. Spatial heterogeneities in calcium homeostasis creates a substrate for action potential duration (APD) alternans, wavebreak, and AF [44,45]. L-type calcium channel antagonists have a relatively modest impact on suppressing AF, although they have been shown to reduce AF in some scenarios, such as after cardioversion [46]. Inhibitors of CaMKII are being studied as potential antiarrhythmic interventions in AF [47]. 

Cardiac nitroso-redox imbalances are increasingly linked to AF. This may represent the regional uncoupling of cellular nitric oxide synthase and the production of reactive oxygen species that modulate the signaling pathways. The oxidation of CaMK-II via angiotensin-II increases Ca^2+^ leak from the sarcoplasmic reticulum that increases AF susceptibility in mice [48]. The atrial-specific upregulation of small non-coding RNAs may disrupt neuronal nitric oxide signaling, shorten refractoriness, and predispose individuals to AF [49]. These pathways are being investigated as novel therapeutic targets. General anti-oxidant therapies such as vitamin C and E have not been effective in suppressing AF in randomized trials [50]. Oxidative stress in epicardial adipose tissue may be the mechanism linking obesity with AF and has been shown to cause atrial fibrosis and predispose individuals to AF [22,51].

Abnormal atrial metabolism is a novel mechanistic cascade, which may operate in AF due to rapid atrial rates for prolonged periods of time. Each sinus rhythm beat expends 2% of myocardial adenosine triphosphate (ATP) stores. This mechanism may explain the link between AF and conditions which impact atrial metabolism, including diabetes mellitus, obesity, heart failure, and thyroid abnormalities. Such abnormalities may in turn drive abnormalities in calcium homeostasis, abnormal nitroso-redox state, and electrical and structural remodeling [52]. Therapy should address each of these identified targets, but other specific therapies are currently unclear.

### 4.2. Structural Remodeling

Several structural abnormalities are observed in patients with AF (Figure 2), although it is unclear to what extent these are a cause or effect of AF. In experimental models, AF can be exacerbated by structural remodeling in the form of atrial enlargement, fibrosis, or epicardial fat accumulation. Conversely, AF can accelerate the progression of atrial dilatation and fibrosis.

Left atrial (LA) dilatation is the most clearly identified form of structural remodeling in patients, and is independently correlated with disease progression and outcome [53,54,55,56]. Intriguing GWAS studies have recently identified genetic loci for atrial dilatation [57]. A smaller LA volume index was associated with a lower risk for AF recurrence in the CABANA trial of ablation or pharmacotherapy [58]. Increasing data implicates right atrial enlargement in conferring a worse prognosis after ablation or cardioversion [59,60]. Atrial enlargement provides more tissue for disordered wavelets or drivers, and also correlates with the presence of fibrosis [61]. The lower incidence of AF in African Americans and Asians compared to Caucasians is associated with the smaller size and altered geometry of the left atrium [62] but, again, it is unclear if this is cause or effect. 

Fibrosis is an intensely studied component of atrial structural remodeling, which has been shown in autopsy studies to co-migrate with the presence of AF rather than age *per se* [61]. Fibrosis introduces heterogeneities in electrical repolarization and conduction, which can facilitate multiple wavelet re-entry or anchor driver regions in optical mapping studies of human AF [63]. It remains unclear how best to quantify fibrosis clinically, although groups have used signal intensity on gadolinium-enhanced magnetic resonance imaging [64] and low-amplitude electrograms in electrophysiology study [65].

Pericardial fat comprises epicardial adipose tissue (EAT), which lies between the visceral pericardium and the epicardium, and paracardial adipose tissue, which lies outside the visceral pericardium. EAT may secrete adipokines, inflammatory cytokines, and reactive oxygen species leading to fibrosis [66]. In the Framingham Heart cohort of 3217 participants, the pericardial fat volume quantified by computed tomography was independently associated with AF. EAT volume is associated with incident persistent AF, with recurrent AF after cardioversion, and potentially with recurrent AF after ablation [67,68]. Although show salutary effects on AF from weight loss have been shown in animal models [69], and left atrial adipose tissue attenuation is associated with human AF recurrence [70], further studies are needed. Weight loss can reduce the symptoms and severity of AF in patients [31], although this was not shown in the LOOK-AHEAD trial of 5067 diabetics [32], or in the recent SORT-AF trial of 133 patients undergoing ablation [33].

### 4.3. Autonomic Remodeling

The heart is richly supplied by the parasympathetic nervous system (via the vagus nerve) and by the cervical sympathetic chain. In animal models, autonomic modulation has been shown to produce early or late after-depolarizations that create triggers or sustain AF. Clinically, the ablation of ganglionated plexus regions has had mixed success in eliminating AF [71], but there has been some success in ablating the renal autonomic ganglia [72]. Another approach is to non-invasively apply low-level vagal nerve stimulation to the tragus of the ear to modulate autonomics rather than denervate the heart [73,74]. Stimulus strengths lower than those which slow the sinus node were shown to modestly reduce AF burden in patients with paroxysmal AF in the TREAT-AF trial [73].

## 5. AF Pathophysiology within the Heart

Identifying the locations of AF mechanisms in the whole heart could enable the spatial targeting of ablation, surgical therapy, pacing, or novel modalities such as external beam irradiation [75]. The recent Early Treatment of Atrial Fibrillation for Stroke Prevention Trial (EAST) Atrial Fibrillation Network (AFNET)-4 trial showed that the early maintenance of sinus rhythm reduces major adverse events [76]. Ablation is more effective at maintaining sinus rhythm than pharmacologic therapy [11], but it needs to improve. Briefly, the success of ablation focused on pulmonary vein isolation (PVI) at 12–18 months ranges from 50–60% for patients with persistent AF [77] to 65–75% for those with PAF using state-of-the-art contact sensing and cryoablation technologies [11,15,78]. The complex physiology, structure, and innervation of PVs may explain their contribution to AF, one that likely extends beyond PVs as source of ectopic triggers [79]. A substantial number of patients have success after AF ablation despite PV reconnection, while many patients with fully isolated PVs have recurrent AF [80,81,82,83]. The identification of additional spatial regions to modify in patients who fail PVI is thus of the utmost importance.

### 5.1. Triggers

AF commences from sinus rhythm through triggers, typically premature beats, as do other supraventricular arrhythmias. Unlike other arrhythmias, AF triggers have a predilection for the pulmonary vein regions of the left atrium. Once initiated, AF is maintained by a series of mechanisms which are less well defined, but again likely comprise electrophysiological, structural, and autonomic factors in each individual patient (Figure 2).

Haïssaguerre et al. reported in 1998 that ectopic impulses near the PVs can trigger paroxysmal AF [84]. Myocardial sleeves within PVs [79,85,86,87,88] are the source for such ectopy, which may be facilitated by the transient factors of stretch, ischemia, or autonomic imbalance [86,89,90,91]. Triggers from other areas (non-PV triggers) can arise from diverse regions, including the superior vena cava, coronary sinus, left atrial appendage, ligament of Marshall, crista terminalis, and the left atrial posterior free wall, and may reflect any of the above mechanisms [85,92,93,94,95]. Unfortunately, beyond the PVs, no single trigger site is dominant; for instance, the large multicenter adjunctive-MAZE (aMAZE) trial recently showed that isolation of the left atrial appendage did not convey benefit over PVI alone [77,96].

### 5.2. Which Triggers Initiate AF?

Relatively little research has investigated why some ectopic beats initiate AF while others do not. As an analogy, premature atrial contractions (PACs) that initiate AV nodal re-entrant tachycardia exploit the relative atrial refractoriness in fast versus slow AV nodal tissue. Our group used monophasic action potentials to study refractoriness (similar to ERP) at the PV antra, other left atrial sites, and right atrial sites in patients with and without AF. We found that PACs initiated PAF if they arose near sites where the restitution slope (rate of change with altering rate) of action potential duration (APD) >1. Conversely, PACs at sites with an APD restitution slope <1 did not initiate PAF. Intriguingly, patients with persistent AF typically showed a APD restitution slope <1 near PVs, while those with paroxysmal AF typically showed a APD restitution slope >1 near PVs. This provides one explanation for why the PVs are less critical in persistent AF [97]. Further work has shown that rapid atrial rates unmask abnormalities in calcium handling that may lead to APD alternans [98,99], regional conduction slowing, and AF onset [100]. Others have shown that AF causes electrical remodeling with longer effective refractory periods and slower conduction, predominantly in the PVs, which begets AF [101]. Together, these studies explain why beta-blockers, which ameliorate autonomic influences, slow the heart rate, and flatten APD restitution [102], may prevent AF in some patients [103]. They may also motivate the role of class I agents, which slow atrial conduction [104].

### 5.3. Mechanisms for the Maintenance of AF Once Initiated (Substrate)

There are different schools of thought regarding how AF is maintained once started. The central debate is whether AF is sustained by localized regions in the atria, which would potentially be amenable to ablation, or by spatially non-localized processes. This has pivotal implications for guiding ablation. It is now widely recognized that AF is not spatially uniform within human atria, with marked differences in regional spatial disorganization, rate gradients, spectral gradients between atria, and within each atrium [105,106,107,108,109,110,111,112,113,114,115,116,117,118]. The debate has shifted to the significance of these non-uniformities, and if and how they may be used to guide ablation (Figure 2, right panel). 

The multiwavelet theory posits that fibrillatory wavelets in AF self-replenish due to the collision between unstable spiral waves and wavebreak. This could be facilitated by factors including transmural dissociation between epi- and endo-myocardium [119,120] and percolation theory [121]. This theory was supported in early computational studies by Moe et al. [122] and experimentally by Allessie et al. [123]. Since this theory does not posit any preferred regions of interest, therapy would require widespread debulking of the atrium to be effective.

Driver theory posits that fibrillatory wavelets in AF are generated, at least in part, by localized regions, i.e., “drivers” that may represent different mechanisms. Focal activity or re-entrant activity, the two predominant electrophysiological mechanisms, have been demonstrated as being AF drivers in several studies. Rotational circuits in AF (also termed “rotors”) are sustained by re-entry around an unexcited, yet excitable, core activating too rapidly for the surrounding tissue to keep up, resulting in wavebreak and fibrillatory conduction, as posited and demonstrated by Jalife et al. [124,125,126]. Re-entrant drivers have been demonstrated by optical mapping in human AF [127]. Focal activity has also been shown to drive AF in animal models and patients [128]. AF drivers could be marked by rapid rate or high dominant frequency [111,129,130]. Less defined localized mechanisms include regions of scar that anchor fibrillatory wavelets [131,132], localized autonomic innervation sites [133], and others.

Studies should move towards defining the regions of the atrium that are critical to AF, even if they are sometimes obscured. This would circumvent the uncertainties over whether a mapping epoch of AF is representative of all epochs of AF, and so on. An analogy is the routine ability to detect and interpret coronary stenosis in patients without angina during the procedure. Potential solutions may include, in patients with specific genomic or clinical profiles [36], the identification of sites of conduction slowing in sinus rhythm and/or rapid pacing [105], sites of scar [39], and potentially sites of abnormal repolarization [98] or abnormal electrogram characteristics.

### 5.4. Clinical Mapping of Driver Regions

**Figure 3:**
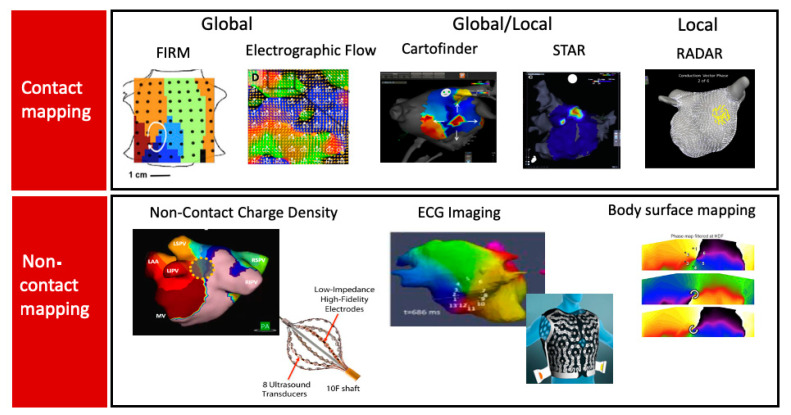
Several mapping systems for atrial fibrillation are now available to guide ablation. Each modality localized regions, typically 3–5 per patient in both atria, that are sufficiently stable in space, although intermittent in time, such that localized ablation may be effective. FIRM shows focal and rotational sites by activation (and phase) annotation. Electrographic flow indicates vectors (here of a rotational site). Cartofinder shows focal (shown) or rotational sites. STAR mapping shows the earliest sites (in warm colors). RADAR shows composite conduction vectors (rotational site shown). Non-contact charge density maps from a non-contact ultrasound-based catheter (illustrated) indicate rotational (shown), focal, or localized irregular activity patterns. ECGI and body surface mapping may reveal rotational (shown) or focal sites using body surface electrodes in proprietary configurations (illustrated for CardioInsight). See text for details and clinical results.

Clinical interest in AF drivers is motivated, in part, by AF drivers identified by optical mapping in human hearts, by clinical observations that limited ablation often terminates persistent AF before PVI is achieved, and by data that AF shows spatial non-uniformities. An increasing variety of tools and methods are available in 2021–2022 to map AF and identify potential drivers. These methods differ in whether signals are recorded by contact or non-contact electrodes, whether the atria are mapped globally or in small regions (locally), and how the signals are processed (Figure 3). It is thus rather surprising, although reassuring, that these divergent systems show many similarities in AF maps: ~3–5 localized regions within disordered AF, showing orderly activation in focal or rotational patterns, in patient-specific locations in either atrium, often outside the pulmonary veins and where ablation can impact or terminate AF in at least some patients.

We summarize the reported AF mapping methods based on whether their primary recording approach is global (panoramic) or small field of view (and hence sequential) in the atria. We also compare mapping systems based on whether they use contact electrodes, which are the gold standard, or non-contact recordings, such as charge density mapping or body surface mapping. These methods are summarized in Figure 3, separated into contact and non-contact approaches.

### 5.5. Contact Mapping 

#### 5.5.1. Global or Panoramic Mapping 

a. Focal Impulse and Rotor Modulation (FIRM) is a prototypical system that maps AF in order to guide ablation. FIRM maps widely within the atria using 64 pole baskets (also used by other systems below), interpreted by activation and phase mapping, that were filtered algorithmically by electrogram features trained to action potential and conduction velocity studies in patients (Rhythmview, Abbott, IL, USA). This approach revealed ~3–5 focal or rotational drivers in each patient, with two-thirds in the left atrium and one-third in the right atrium. Drivers were intermittent yet relatively stable in space for prolonged periods of time. The results of FIRM are ~80% concordant with the concurrent optical mapping of AF in explanted human atria [127], with promising results by targeted ablation in meta-analyses [128,134,135]. The pivotal trial of this approach (REAFFIRM) showed no difference between PVI and PVI plus driver ablation on intention-to-treat analysis. However, a high number of cross-overs between limbs (~50%) diluted its power. On-treatment analysis revealed 77.8% freedom from all atrial arrhythmias by PVI plus driver ablation versus 65.5% for PVI (*p* = 0.08) at 1 year. This hypothesis-generating result has motivated several techniques to map AF. While studies using FIRM included control against PVI alone, reports of newer techniques to date have mostly been single limb. Randomized controlled trials of these approaches are ongoing. Several improved algorithms have been proposed using existing forms of global contact mapping.

b. Electrographic flow mapping uses similar panoramic basket catheter recordings, instead analyzed using the Horn–Schunck optical flow algorithm to calculate the average electrical flow of propagation of action potentials that is proposed to be resistant to noise and artifacts [135]. Applied retrospectively to FIRM data, the approach has been used to identify FIRM regions that may be of higher or lower importance, including sites where targeted ablation terminated AF [136]. The commercially available system, Ablacon (Ablamap^TM^, Ablacon Inc. Wheat Ridge, CO, USA), is currently undergoing prospective evaluation to guide AF ablation [137].

#### 5.5.2. Local Contact Mapping, i.e., Small Regions Mapped Sequentially

a. Spatiotemporal dispersion mapping identifies areas of stable electrogram patterns across the splines of a high-density catheter (Pentaray, Biosense-Webster, Diamond Bar, CA, USA) that span the AF cycle length and represent the electrogram fingerprints of nearby rotational drivers. About 40% of the patients had dispersion areas in the right atrium [138]. These areas were higher in persistent AF than in paroxysmal AF. Targeting these drivers for ablation enabled a 95% acute termination rate and 85% freedom from AF at 18 months in a diverse AF population (paroxysmal AF had better acute and long-term outcomes than long-standing persistent AF) [138,139]. 

b. Stochastic trajectory analysis of ranked signals (STAR) analyzes either global recordings from basket catheters or localized signals from multiple catheters to identify regions in AF that most often precede the activation of the neighboring areas. This is done by creating a statistical model from hundreds of activations, ranking the regions of the atrium by the amount of time that their activations precede those of the adjacent regions. Per patient, 2.6 ± 0.8 early sites of electrical activity (ESA) were identified, 73.8% of which persisted after PVI. One-fourth of the patients (8/32) underwent right atrial mapping, of whom three had one ESA each. Ablation of all sites lengthened cycle-length by ≥30 ms [140]. ESAs resulting in AF termination were more likely to be identified on both pre- and post-PVI maps than on those associated with cycle length slowing (23 of 24 vs. 16 of 49; *p* < 0.001). At 12 months follow-up, 80% of these PsAF patients were free from AF/AT [140].

c. Real-Time Electrogram Analysis for Drivers of Atrial Fibrillation (RADAR): using the coronary sinus as a reference, this system sorts and compiles electrograms recorded in small regions using a standard mapping catheter. An elegant approach bins localized recordings at several hundred locations with a similar coronary sinus electrogram pattern into one global map to calculate 3-D conduction vectors, then a driver density map. This is repeated for all observed coronary sinus patterns. Multiple maps are fused probabilistically based on the repetition of rotational or focal drivers at the border of low-voltage areas to highlight putative AF drivers, which are targeted for ablation. A total of 5% of de novo and 23% of redo ablation patients had right atrial drivers in this study, with an average of 2.5 drivers per patient [141]. Initial results from ablation using this approach in a population of 64 patients showed 74% freedom from arrhythmias on/off drug (and 68% off drug) at 13 months follow-up [141]. 

#### 5.5.3. Mixed (Both Local and Global)

a. Cartofinder uses combined unipolar and bipolar electrogram annotation to construct high-density activation maps using either a panoramic basket catheter [142] or a high-density localized catheter (Biosense-Webster, CA, USA) in recent series [143]. Focal drivers are more common with this approach than rotational drivers, with 82% reproducibility, of which 55% following PVI, motivating the need to ablate areas beyond PVI [142]. About 7% of focal and 4% of rotational activations were seen in the right lateral area (including the right atrium) with this approach [143]. Ablating these areas was associated with higher acute termination rates than PVI alone (75% vs. 38%, *p* = 0.006) [143]. However, 47% of patients undergoing such ablation recurred on median follow-up of 531 days [144]. 

#### 5.5.4. Non-Contact Mapping

a. Non-contact charge density mapping: this approach is based on the physical principle that the membrane charge layer is the true source of the cardiac field, and therefore, the calculated charge density provides the most accurate localization of drivers. Mapping is done with a specialized non-contact catheter with 48 ultrasound emitters and electrodes. The ultrasound emitters are used in real time to generate a 3D anatomy by rotation of the assembly in the center of the atrial chamber, and unipolar electrograms (150 k s^−1^) acquired by the electrodes are used to calculate the charge density at fixed times using a governing Poisson formulation. This is displayed as a movie on a dedicated console (Acutus Inc, Carlsbad, CA, USA) [145,146]. With caveats that validation against contact electrograms may be modest in AF, especially in larger atria [147], the system identifies localized rotational activity, focal beats, and localized irregular activity in AF. Ablation at these areas has shown 72.3% freedom from AF at 12 months with an index procedure that combined AF trigger mapping and ablation with PVI [148].

b. Electrocardiographic Imaging (ECGi): using an inverse solution to reconstruct biatrial unipolar electrograms from torso potentials acquired using a 252-electrode surface vest (ECVUE, Cardioinsight, Medtronic, Palo Alto, CA, USA) and a non-contrast thoracic computed tomography scan, activation maps are computed using the traditional unipolar electrogram intrinsic deflection-based [(−dV/dT) max] method. Movies of activation and/or phase are then used to show “driver domains” biatrially, which can then be used to guide ablation to reduce the complexity of AF to atrial tachycardias. Of all drivers, 28% were found in the right atrium in the AFACART study [149]. This approach has been associated with higher freedom from AF compared to historical stepwise ablation cohorts [150], although with up to a 50% recurrence rate of atrial tachycardia [149].

## 6. Conclusions

AF can progress from a disease with sporadic episodes, relat to mechanisms near the pulmonary veins, to a persistent disease encompassing mechanisms at genetic, cellular, organ, and patient levels. This argues strongly against the use of “one size fits all” therapies which, indeed, have had modest success in clinical trials. To personalize ablation, it seems increasingly necessary to map AF in each patient to identify the non-stereotypical targets. Contemporary AF mapping tools must be improved to realize this goal, although several approaches show promise. To personalize therapy more broadly, it is necessary to consider the nuanced relationship between the clinical, demographic, metabolic, and genomic mechanisms for each patient. Future tailored approaches may integrate mechanistic markers at these biological levels, which could be achieved using machine learning to develop individualized models of AF onset, progression, and response to therapy. This exciting goal for precision medicine is increasingly tractable, and we look forward to further developments in this field.

## References

[1] Chugh S.S., Havmoeller R., Narayanan K., Singh D., Rienstra M., Benjamin E.J., Gillum R.F., Kim Y.H., McAnulty J.H., Zheng Z.J. (2014). Worldwide epidemiology of atrial fibrillation: A Global Burden of Disease 2010 Study. Circulation.

[2] Lane D.A., Skjøth F., Lip G.Y.H., Larsen T.B., Kotecha D. (2017). Temporal Trends in Incidence, Prevalence, and Mortality of Atrial Fibrillation in Primary Care. J. Am. Heart Assoc..

[3] Savelieva I., Camm A.J. (2000). Clinical relevance of silent atrial fibrillation: Prevalence, prognosis, quality of life, and management. J. Interv. Card. Electrophysiol..

[4] Olshansky B., Knight B.P., Yeon S.B. (2021). The electrocardiogram in atrial fibrillation. UpToDate.

[5] Krittanawong C., Johnson K.W., Rosenson R.S., Wang Z., Aydar M., Baber U., Min J.K., Tang W.W., Halperin J.L., Narayan S.M. (2019). Deep learning for cardiovascular medicine: A practical primer. Eur. Heart J..

[6] Rogers A.J., Selvalingam A., Alhusseini M.I., Krummen D.E., Corrado C., Abuzaid F., Baykaner T., Meyer C., Clopton P., Giles W. (2021). Machine Learned Cellular Phenotypes in Cardiomyopathy Predict Sudden Death. Circ. Res..

[7] Nattel S., Dobrev D. (2016). Deciphering the fundamental mechanisms of atrial fibrillation: A quest for over a century. Cardiovasc. Res..

[8] Peirlinck M., Costabal F.S., Yao J., Guccione J.M., Tripathy S., Wang Y., Ozturk D., Segars P., Morrison T.M., Levine S. (2021). Precision medicine in human heart modeling: Perspectives, challenges, and opportunities. Biomech. Model. Mechanobiol..

[9] Calkins H., Hindricks G., Cappato R., Kim Y.H., Saad E.B., Aguinaga L., Akar J.G., Badhwar V., Brugada J., Camm J. (2017). 2017 HRS/EHRA/ECAS/APHRS/SOLAECE Expert Consensus Statement on Catheter and Surgical Ablation of Atrial Fibrillation. Heart Rhythm..

[10] Charitos E.I., Pürerfellner H., Glotzer T.V., Ziegler P.D. (2014). Clinical classifications of atrial fibrillation poorly reflect its temporal persistence: Insights from 1195 patients continuously monitored with implantable devices. J. Am. Coll. Cardiol..

[11] Poole J.E., Bahnson T.D., Monahan K.H., Johnson G., Rostami H., Silverstein A.P., Al-Khalidi H.R., Rosenberg Y., Mark D.B., Lee K.L. (2020). Recurrence of Atrial Fibrillation After Catheter Ablation or Antiarrhythmic Drug Therapy in the CABANA Trial. J. Am. Coll. Cardiol..

[12] Andrade J.G., Yao R.R., Deyell M.W., Hawkins N.M., Rizkallah J., Jolly U., Khoo C., Raymond J.M., McKinney J., Cheung C. (2020). Clinical assessment of AF pattern is poorly correlated with AF burden and post ablation outcomes: A CIRCA-DOSE sub-study. J. Electrocardiol..

[13] Jons C., Hansen P.S., Johannessen A., Hindricks G., Raatikainen P., Kongstad O., Walfridsson H., Pehrson S., Almroth H., Hartikainen J. (2009). The Medical ANtiarrhythmic Treatment or Radiofrequency Ablation in Paroxysmal Atrial Fibrillation (MANTRA-PAF) trial: Clinical rationale, study design, and implementation. Europace.

[14] Marrouche N.F., Brachmann J., Andresen D., Siebels J., Boersma L., Jordaens L., Merkely B., Pokushalov E., Sanders P., Proff J. (2018). Catheter Ablation for Atrial Fibrillation with Heart Failure. N. Engl. J. Med..

[15] Wazni O.M., Dandamudi G., Sood N., Hoyt R., Tyler J., Durrani S., Niebauer M., Makati K., Halperin B., Gauri A. (2020). Cryoballoon Ablation as Initial Therapy for Atrial Fibrillation. N. Engl. J. Med..

[16] Tucker N.R., Clauss S., Ellinor P.T. (2016). Common variation in atrial fibrillation: Navigating the path from genetic association to mechanism. Cardiovasc. Res..

[17] Packer M. (2020). Characterization, Pathogenesis, and Clinical Implications of Inflammation-Related Atrial Myopathy as an Important Cause of Atrial Fibrillation. J. Am. Heart Assoc..

[18] Linz D., McEvoy R.D., Cowie M.R., Somers V.K., Nattel S., Lévy P., Kalman J.M., Sanders P. (2018). Associations of Obstructive Sleep Apnea With Atrial Fibrillation and Continuous Positive Airway Pressure Treatment: A Review. JAMA Cardiol..

[19] Voskoboinik A., Kalman J.M., De Silva A., Nicholls T., Costello B., Nanayakkara S., Prabhu S., Stub D., Azzopardi S., Vizi D. (2020). Alcohol Abstinence in Drinkers with Atrial Fibrillation. N. Engl. J. Med..

[20] Guasch E., Benito B., Qi X., Cifelli C., Naud P., Shi Y., Mighiu A., Tardif J.C., Tadevosyan A., Chen Y. (2013). Atrial fibrillation promotion by endurance exercise: Demonstration and mechanistic exploration in an animal model. J. Am. Coll. Cardiol..

[21] Wang N., Sun Y., Zhang H., Wang B., Chen C., Wang Y., Chen J., Tan X., Zhang J., Xia F. (2021). Long-term night shift work is associated with the risk of atrial fibrillation and coronary heart disease. Eur. Heart J..

[22] Wang T.J., Parise H., Levy D., D’Agostino R.B., Wolf P.A., Vasan R.S., Benjamin E.J. (2004). Obesity and the Risk of New-Onset Atrial Fibrillation. JAMA.

[23] Wang T.J., Larson M.G., Levy D., Vasan R.S., Leip E.P., Wolf P.A., D’Agostino R.B., Murabito J.M., Kannel W.B., Benjamin E.J. (2003). Inter-Relationship between AF and CHF in the Framingham Heart Study. Circulation.

[24] Barrett T.W., Self W.H., Wasserman B.S., McNaughton C.D., Darbar D. (2013). Evaluating the HATCH score for predicting progression to sustained atrial fibrillation in ED patients with new atrial fibrillation. Am. J. Emerg. Med..

[25] Potpara T.S., Polovina M.M., Marinkovic J.M., Lip G.Y. (2013). A comparison of clinical characteristics and long-term prognosis in asymptomatic and symptomatic patients with first-diagnosed atrial fibrillation: The Belgrade Atrial Fibrillation Study. Int. J. Cardiol..

[26] Lip G.Y.H., Skjoth F., Nielsen P.B., Larsen T.B. (2020). Evaluation of the C2HEST Risk Score as a Possible Opportunistic Screening Tool for Incident Atrial Fibrillation in a Healthy Population (From a Nationwide Danish Cohort Study). Am. J. Cardiol..

[27] Khurshid S., Kartoun U., Ashburner J.M., Trinquart L., Philippakis A., Khera A.V., Ellinor P.T., Ng K., Lubitz S.A. (2021). Performance of Atrial Fibrillation Risk Prediction Models in Over 4 Million Individuals. Circ. Arrhythm. Electrophysiol..

[28] Kornej J., Hindricks G., Kosiuk J., Arya A., Sommer P., Husser D., Rolf S., Richter S., Huo Y., Piorkowski C. (2014). Comparison of CHADS2, R2CHADS2, and CHA2DS2-VASc scores for the prediction of rhythm outcomes after catheter ablation of atrial fibrillation: The Leipzig Heart Center AF Ablation Registry. Circ. Arrhythm. Electrophysiol..

[29] Kornej J., Hindricks G., Shoemaker M.B., Husser D., Arya A., Sommer P., Rolf S., Saavedra P., Kanagasundram A., Whalen S.P. (2015). The APPLE score: A novel and simple score for the prediction of rhythm outcomes after catheter ablation of atrial fibrillation. Clin. Res. Cardiol..

[30] van den Ham H.A., Klungel O.H., Singer D.E., Leufkens H.G., van Staa T.P. (2015). Comparative Performance of ATRIA, CHADS2, and CHA2DS2-VASc Risk Scores Predicting Stroke in Patients With Atrial Fibrillation: Results From a National Primary Care Database. J. Am. Coll. Cardiol..

[31] Abed H.S., Wittert G.A., Leong D.P., Shirazi M.G., Bahrami B., Middeldorp M.E., Lorimer M.F., Lau D.H., Antic N.A., Brooks A.G. (2013). Effect of weight reduction and cardiometabolic risk factor management on symptom burden and severity in patients with atrial fibrillation: A randomized clinical trial. JAMA.

[32] Alonso A., Bahnson J.L., Gaussoin S.A., Bertoni A., Johnson K.C., Lewis C.E., Vetter M., Mantzoros C.S., Jeffery R.W., Soliman E.Z. (2015). Effect of an intensive lifestyle intervention on atrial fibrillation risk in individuals with type 2 diabetes: The Look AHEAD randomized trial. Am. Heart J..

[33] Gessler N., Willems S., Steven D., Aberle J., Akbulak R.O., Gosau N., Hoffmann B.A., Meyer C., Sultan A., Tilz R. (2021). Supervised Obesity Reduction Trial for AF Ablation Patients: Results from the SORT-AF trial. Europace.

[34] Mahida S., Lubitz S.A., Rienstra M., Milan D.J., Ellinor P.T. (2011). Monogenic atrial fibrillation as pathophysiological paradigms. Cardiovasc. Res..

[35] Yoneda Z.T., Anderson K.C., Quintana J.A., O’Neill M.J., Sims R.A., Glazer A.M., Shaffer C.M., Crawford D.M., Stricker T., Ye F. (2021). Early-Onset Atrial Fibrillation and the Prevalence of Rare Variants in Cardiomyopathy and Arrhythmia Genes. JAMA Cardiol..

[36] Nielsen J.B., Thorolfsdottir R.B., Fritsche L.G., Zhou W., Skov M.W., Graham S.E., Herron T.J., McCarthy S., Schmidt E., Sveinbjornsson G. (2018). Biobank-driven genomic discovery yields new insight into atrial fibrillation biology. Nat. Genet..

[37] Shoemaker M.B., Husser D., Roselli C., Al Jazairi M., Chrispin J., Kühne M., Neumann B., Knight S., Sun H., Mohanty S. (2020). Genetic Susceptibility for Atrial Fibrillation in Patients Undergoing Atrial Fibrillation Ablation. Circ. Arrhythm. Electrophysiol..

[38] Unterhuber M., Kresoja K.-P., Rommel K.-P., Besler C., Baragetti A., Klöting N., Ceglarek U., Blüher M., Scholz M., Catapano A.L. (2021). Proteomics-Enabled Deep Learning Machine Algorithms Can Enhance Prediction of Mortality. J. Am. Coll. Cardiol..

[39] Marrouche N.F., Wilber D., Hindricks G., Jais P., Akoum N., Marchlinski F., Kholmovski E., Burgon N., Hu N., Mont L. (2014). Association of atrial tissue fibrosis identified by delayed enhancement MRI and atrial fibrillation catheter ablation: The DECAAF study. JAMA.

[40] Heijman J., Voigt N., Nattel S., Dobrev D. (2014). Cellular and molecular electrophysiology of atrial fibrillation initiation, maintenance, and progression. Circ. Res..

[41] Li D., Benardeau A., Nattel S. (2000). Contrasting efficacy of dofetilide in different experimental models of atrial fibrillatoin. Circulation.

[42] Chaldoupi S.M., Loh P., Hauer R.N., de Bakker J.M., van Rijen H.V. (2009). The role of connexin40 in atrial fibrillation. Cardiovasc. Res..

[43] Voigt N., Heijman J., Wang Q., Chiang D.Y., Li N., Karck M., Wehrens X.H.T., Nattel S., Dobrev D. (2014). Cellular and molecular mechanisms of atrial arrhythmogenesis in patients with paroxysmal atrial fibrillation. Circulation.

[44] Zlochiver S., Johnson C., Tolkacheva E.G. (2017). Constant DI pacing suppresses cardiac alternans formation in numerical cable models. Chaos.

[45] Renard E., Surget E., Michel C., Benoist D., Dubes V., Walton R.D., Martinez M.E., Guillot B., Constantin M., Hocini M. (2021). B-AB01-01 local repolarization heterogeneity is more arrhythmogenic than structural and functional conduction heterogeneities in an ex vivo porcine model. Heart Rhythm..

[46] Roux J.F., Zado E., Callans D.J., Garcia F., Lin D., Marchlinski F., Bala R., Dixit S., Riley M., Russo A.M. (2009). Antiarrhythmics After Ablation of Atrial Fibrillation (5A Study). Circulation.

[47] Mesubi O.O., Anderson M.E. (2016). Atrial remodelling in atrial fibrillation: CaMKII as a nodal proarrhythmic signal. Cardiovasc. Res..

[48] Purohit A., Rokita A.G., Guan X., Chen B., Koval O., Voigt N., Neef S., Sowa T., Gao Z., Luczak E.D. (2013). Oxidized Ca(2+)/calmodulin-dependent protein kinase II triggers atrial fibrillation. Circulation.

[49] Reilly S.N., Liu X., Carnicer R., Recalde A., Muszkiewicz A., Jayaram R., Carena M.C., Wijesurendra R., Stefanini M., Surdo N.C. (2016). Up-regulation of miR-31 in human atrial fibrillation begets the arrhythmia by depleting dystrophin and neuronal nitric oxide synthase. Sci. Transl. Med..

[50] Violi F., Pastori D., Pignatelli P., Loffredo L. (2014). Antioxidants for prevention of atrial fibrillation: A potentially useful future therapeutic approach? A review of the literature and meta-analysis. Europace.

[51] Abed H.S., Samuel C.S., Lau D.H., Kelly D., Royce S.G., Alasady M., Mahajan R., Kuklik P., Zhang Y., Brooks A.G. (2013). Obesity results in progressive atrial structural and electrical remodeling: Implications for atrial fibrillation. Heart Rhythm..

[52] Opacic D., van Bragt K.A., Nasrallah H.M., Schotten U., Verheule S. (2016). Atrial metabolism and tissue perfusion as determinants of electrical and structural remodelling in atrial fibrillation. Cardiovasc. Res..

[53] Olshansky B., Heller E.N., Mitchell L.B., Chandler M., Slater W., Green M., Brodsky M., Barrell P., Greene H.L. (2005). Are Transthoracic Echocardiographic Parameters Associated With Atrial Fibrillation Recurrence or Stroke?: Results From the Atrial Fibrillation Follow-Up Investigation of Rhythm Management (AFFIRM) Study. J. Am. Coll. Cardiol..

[54] Bunch T.J., Munger T.M., Friedman P.A., Asirvatham S.J., Brady P.A., Cha Y.-M., Rea R.F., Shen W.-K., Powell B.D., Ommen S.R. (2008). Substrate and procedural predictors of outcomes after catheter ablation for atrial fibrillation in patients with hypertrophic cardiomyopathy. J. Cardiovasc. Electrophysiol..

[55] Njoku A., Kannabhiran M., Arora R., Reddy P., Gopinathannair R., Lakkireddy D., Dominic P. (2018). Left atrial volume predicts atrial fibrillation recurrence after radiofrequency ablation: A meta-analysis. Europace.

[56] Demarchi A., Neumann L., Rordorf R., Conte G., Sanzo A., Özkartal T., Savastano S., Regoli F., Vicentini A., Caputo M.L. (2021). Long-term outcome of catheter ablation for atrial fibrillation in patients with severe left atrial enlargement and reduced left ventricular ejection fraction. Europace.

[57] Ahlberg G., Andreasen L., Ghouse J., Bertelsen L., Bundgaard H., Haunsø S., Svendsen J.H., Olesen M.S. (2021). Genome-wide association study identifies 18 novel loci associated with left atrial volume and function. Eur. Heart J..

[58] Rettmann M.E., Holmes D.R., Monahan K.H., Breen J.F., Bahnson T.D., Mark D.B., Poole J.E., Ellis A.M., Silverstein A.P., Al-Khalidi H.R. (2021). Treatment-Related Changes in Left Atrial Structure in Atrial Fibrillation: Findings From the CABANA Imaging Substudy. Circ. Arrhythm. Electrophysiol..

[59] Luong C., Thompson D.J., Bennett M., Gin K., Jue J., Barnes M.E., Colley P., Tsang T.S. (2015). Right atrial volume is superior to left atrial volume for prediction of atrial fibrillation recurrence after direct current cardioversion. Can. J. Cardiol..

[60] Johner N., Namdar M., Shah D.C. (2020). Right Atrial Complexity Evolves With Stepwise Left-Sided Persistent Atrial Fibrillation Substrate Ablation and Predicts Outcomes. JACC Clin. Electrophysiol..

[61] Platonov P.G., Mitrofanova L.B., Orshanskaya V., Ho S.Y. (2011). Structural abnormalities in atrial walls are associated with presence and persistency of atrial fibrillation but not with age. J. Am. Coll. Cardiol..

[62] Lau C.P., Gbadebo T.D., Connolly S.J., Van Gelder I.C., Capucci A., Gold M.R., Israel C.W., Morillo C., Siu C.-W., Abe H. (2013). Ethnic differences in atrial fibrillation identified using implanted cardiac devices. J. Cardiovasc. Electrophysiol..

[63] Zahid S., Whyte K.N., Schwarz E.L., Blake R.C., Boyle P.M., Chrispin J., Prakosa A., Ipek E.G., Pashakhanloo F., Halperin H.R. (2016). Feasibility of using patient-specific models and the “minimum cut” algorithm to predict optimal ablation targets for left atrial flutter. Heart Rhythm..

[64] Marrouche N.F., Greene T., Dean J.M., Kholmovski E.G., Boer L.M., Mansour M., Calkins H., Marchlinski F., Wilber D., Hindricks G. (2021). Efficacy of LGE-MRI-guided fibrosis ablation versus conventional catheter ablation of atrial fibrillation: The DECAAF II trial: Study design. J. Cardiovasc. Electrophysiol..

[65] Zghaib T., Keramati A., Chrispin J., Huang D., Balouch M.A., Ciuffo L., Berger R.D., Marine J.E., Ashikaga H., Calkins H. (2018). Multimodal Examination of Atrial Fibrillation Substrate: Correlation of Left Atrial Bipolar Voltage Using Multi-Electrode Fast Automated Mapping, Point-by-Point Mapping, and Magnetic Resonance Image Intensity Ratio. JACC Clin. Electrophysiol..

[66] Venteclef N., Guglielmi V., Balse E., Gaborit B., Cotillard A., Atassi F., Amour J., Leprince P., Dutour A., Clément K. (2013). Human epicardial adipose tissue induces fibrosis of the atrial myocardium through the secretion of adipo-fibrokines. Eur. Heart J..

[67] Stojanovska J., Kazerooni E.A., Sinno M., Gross B.H., Watcharotone K., Patel S., Jacobson J.A., Oral H. (2015). Increased epicardial fat is independently associated with the presence and chronicity of atrial fibrillation and radiofrequency ablation outcome. Eur. Radiol..

[68] Hatem S.N., Redheuil A., Gandjbakhch E. (2016). Cardiac adipose tissue and atrial fibrillation: The perils of adiposity. Cardiovasc. Res..

[69] Mahajan R., Lau D.H., Brooks A.G., Shipp N.J., Wood J.P., Manavis J., Samuel C.S., Patel K.P., Finnie J.W., Alasady M. (2021). Atrial Fibrillation and Obesity: Reverse Remodeling of Atrial Substrate With Weight Reduction. JACC Clin. Electrophysiol..

[70] El Mahdiui M., Simon J., Smit J.M., Kuneman J.H., van Rosendael A.R., Steyerberg E.W., van der Geest R.J., Száraz L., Herczeg S., Szegedi N. (2021). Posterior Left Atrial Adipose Tissue Attenuation Assessed by Computed Tomography and Recurrence of Atrial Fibrillation after Catheter Ablation. Circ. Arrhythm. Electrophysiol..

[71] Katritsis D.G., Pokushalov E., Romanov A., Giazitzoglou E., Siontis G.C., Po S.S., Camm A.J., Ioannidis J.P. (2013). Autonomic denervation added to pulmonary vein isolation for paroxysmal atrial fibrillation: A randomized clinical trial. J. Am. Coll. Cardiol..

[72] Steinberg J.S., Shabanov V., Ponomarev D., Losik D., Ivanickiy E., Kropotkin E., Polyakov K., Ptaszynski P., Keweloh B., Yao C.J. (2020). Effect of Renal Denervation and Catheter Ablation vs Catheter Ablation Alone on Atrial Fibrillation Recurrence Among Patients With Paroxysmal Atrial Fibrillation and Hypertension: The ERADICATE-AF Randomized Clinical Trial. JAMA.

[73] Stavrakis S., Stoner J.A., Humphrey M.B., Morris L., Filiberti A., Reynolds J.C., Elkholey K., Javed I., Twidale N., Riha P. (2020). TREAT AF (Transcutaneous Electrical Vagus Nerve Stimulation to Suppress Atrial Fibrillation): A Randomized Clinical Trial. JACC Clin. Electrophysiol..

[74] Kulkarni K., Singh J.P., Parks K.A., Katritsis D.G., Stavrakis S., Armoundas A.A. (2021). Low-Level Tragus Stimulation Modulates Atrial Alternans and Fibrillation Burden in Patients With Paroxysmal Atrial Fibrillation. J. Am. Heart Assoc..

[75] Chiu M.H., Mitchell L.B., Ploquin N., Faruqi S., Kuriachan V.P. (2021). Review of Stereotactic Arrhythmia Radioablation Therapy for Cardiac Tachydysrhythmias. CJC Open.

[76] Kirchhof P., Camm A.J., Goette A., Brandes A., Eckardt L., Elvan A., Fetsch T., Van Gelder I.C., Haase D., Haegeli L.M. (2020). Early Rhythm-Control Therapy in Patients with Atrial Fibrillation. N. Engl. J. Med..

[77] Clarnette J.A., Brooks A.G., Mahajan R., Elliott A.D., Twomey D., Pathak R.K., Kumar S., Munawar D.A., Young G.D., Kalman J.M. (2018). Outcomes of persistent and long-standing persistent atrial fibrillation ablation: A systematic review and meta-analysis. Europace.

[78] Andrade J.G., Wells G.A., Deyell M.W., Bennett M., Essebag V., Champagne J., Roux J.-F., Yung D., Skanes A., Khaykin Y. (2020). Cryoablation or Drug Therapy for Initial Treatment of Atrial Fibrillation. N. Engl. J. Med..

[79] Khan R. (2004). Identifying and understanding the role of pulmonary vein activity in atrial fibrillation. Cardiovasc. Res..

[80] Nery P.B., Belliveau D., Nair G.M., Bernick J., Redpath C.J., Szczotka A., Sadek M.M., Green M.S., Wells G., Birnie D.H. (2016). Relationship Between Pulmonary Vein Reconnection and Atrial Fibrillation Recurrence: A Systematic Review and Meta-Analysis. JACC Clin. Electrophysiol..

[81] Pratola C., Baldo E., Notarstefano P., Toselli T., Ferrari R. (2008). Radiofrequency atrial fibrillation ablation based on pathophysiology: A diversified protocol with long-term follow-up. J. Cardiovasc. Med..

[82] Kuck K.H., Hoffmann B.A., Ernst S., Wegscheider K., Treszl A., Metzner A., Eckardt L., Lewalter T., Breithardt G., Willems S. (2016). Impact of Complete Versus Incomplete Circumferential Lines Around the Pulmonary Veins During Catheter Ablation of Paroxysmal Atrial Fibrillation: Results From the Gap-Atrial Fibrillation-German Atrial Fibrillation Competence Network 1 Trial. Circ. Arrhythm. Electrophysiol..

[83] Prabhu S., Kalla M., Peck K.Y., Voskoboinik A., McLellan A.J., Pathik B., Nalliah C.J., Wong G.R., Sugumar H., Azzopardi S.M. (2018). Pulmonary vein activity does not predict the outcome of catheter ablation for persistent atrial fibrillation: A long-term multicenter prospective study. Heart Rhythm..

[84] Haissaguerre M., Jaïs P., Shah D.C., Takahashi A., Hocini M., Quiniou G., Garrigue S., Le Mouroux A., Le Métayer P., Clémenty J. (1998). Spontaneous Initiation of Atrial Fibrillation by Ectopic Beats Originating in the Pulmonary Veins. N. Engl. J. Med..

[85] Chen S.-A., Hsieh M.H., Tai C.T., Tsai C.F., Prakash V.S., Yu W.C., Hsu T.L., Ding Y.A., Chang M.S. (1999). Initiation of Atrial Fibrillation by Ectopic Beats Originating From the Pulmonary Veins: Electrophysiological Characteristics, Pharmacological Responses, and Effects of Radiofrequency Ablation. Circulation.

[86] Hocini M., Ho S.Y., Kawara T., Linnenbank A.C., Potse M., Shah D., Jaïs P., Janse M.J., Haïssaguerre M., De Bakker J.M. (2002). Electrical conduction in canine pulmonary veins: Electrophysiological and anatomic correlation. Circulation.

[87] Yamane T., Shah D.C., Jaïs P., Hocini M., Deisenhofer I., Choi K.-J., Macle L., Clémenty J., Haïssaguerre M. (2002). Electrogram polarity reversal as an additional indicator of breakthroughs from the left atrium to the pulmonary veins. J. Am. Coll. Cardiol..

[88] Patterson E., Lazzara R., Szabo B., Liu H., Tang D., Li Y.-H., Scherlag B.J., Po S.S. (2006). Sodium-calcium exchange initiated by the Ca2+ transient: An arrhythmia trigger within pulmonary veins. J. Am. Coll. Cardiol..

[89] Yamazaki M., Vaquero L.M., Hou L., Campbell K., Zlochiver S., Klos M., Mironov S., Berenfeld O., Honjo H., Kodama I. (2009). Mechanisms of stretch-induced atrial fibrillation in the presence and the absence of adrenocholinergic stimulation: Interplay between rotors and focal discharges. Heart Rhythm..

[90] Wijesurendra R.S., Casadei B. (2019). Mechanisms of atrial fibrillation. Heart.

[91] Jais P., Hocini M., Macle L., Choi K.-J., Deisenhofer I., Weerasooriya R., Shah D.C., Garrigue S., Raybaud F., Scavee C. (2002). Distinctive Electrophysiological Properties of Pulmonary Veins in Patients With Atrial Fibrillation. Circulation.

[92] Wit A.L., Cranefield P.F. (1977). Triggered and automatic activity in the canine coronary sinus. Circ. Res..

[93] Schmitt C., Ndrepepa G., Weber S., Schmieder S., Weyerbrock S., Schneider M., Karch M.R., Deisenhofer I., Schreieck J., Zrenner B. (2002). Biatrial multisite mapping of atrial premature complexes triggering onset of atrial fibrillation. Am. J. Cardiol..

[94] Lin W.-S., Tai C.-T., Hsieh M.-H., Tsai C.-F., Lin Y.-K., Tsao H.-M., Huang J.-L., Yu W.-C., Yang S.-P., Ding Y.-A. (2003). Catheter ablation of paroxysmal atrial fibrillation initiated by non-pulmonary vein ectopy. Circulation.

[95] Santangeli P., Marchlinski F.E. (2017). Techniques for the provocation, localization, and ablation of non-pulmonary vein triggers for atrial fibrillation. Heart Rhythm..

[96] Li F., Sun J.-Y., Wu L.-D., Hao J.-F., Wang R.-X. (2021). The long-term efficacy and safety of combining ablation and left atrial appendage closure: A systematic review and meta-analysis. J. Cardiovasc. Electrophysiol..

[97] Narayan S.M., Kazi D., Krummen D.E., Rappel W.-J. (2008). Repolarization and Activation Restitution Near Human Pulmonary Veins and Atrial Fibrillation Initiation: A Mechanism for the Initiation of Atrial Fibrillation by Premature Beats. J. Am. Coll. Cardiol..

[98] Narayan S.M., Franz M.R., Clopton P., Pruvot E.J., Krummen D.E. (2011). Repolarization Alternans Reveals Vulnerability to Human Atrial Fibrillation. Circulation.

[99] Krummen D.E., Bayer J.D., Ho J., Ho G., Smetak M.R., Clopton P., Trayanova N.A., Narayan S.M. (2012). Mechanisms of human atrial fibrillation initiation: Clinical and computational studies of repolarization restitution and activation latency. Circ. Arrhythm. Electrophysiol..

[100] Lalani G., Schricker A., Gibson M., Rostamian A., Krummen D.E., Narayan S.M. (2012). Atrial Conduction Slows Immediately Before the Onset of Human Atrial Fibrillation: A Bi-Atrial Contact Mapping Study of Transitions to Atrial Fibrillation. J. Am. Coll. Cardiol..

[101] Rostock T., Steven D., Lutomsky B., Servatius H., Drewitz I., Klemm H., Müllerleile K., Ventura R., Meinertz T., Willems S. (2008). Atrial fibrillation begets atrial fibrillation in the pulmonary veins on the impact of atrial fibrillation on the electrophysiological properties of the pulmonary veins in humans. J. Am. Coll. Cardiol..

[102] Hao S.C., Christini D.J., Stein K.M., Jordan P.N., Iwai S., Bramwell O., Markowitz S.M., Mittal S., Lerman B.B. (2004). Effect of beta-adrenergic blockade on dynamic electrical restitution in vivo. Am. J. Physiol. Heart Circ. Physiol..

[103] Karwath A., Bunting K.V., Gill S.K., Tica O., Pendleton S., Aziz F., Barsky A.D., Chernbumroong S., Duan J., Mobley A.R. (2021). Redefining β-blocker response in heart failure patients with sinus rhythm and atrial fibrillation: A machine learning cluster analysis. Lancet.

[104] Shinagawa K., Shiroshita-Takeshita A., Schram G., Nattel S. (2003). Effects of Antiarrhythmic Drugs on Fibrillation in the Remodeled Atrium: Insights Into the Mechanism of the Superior Efficacy of Amiodarone. Circulation.

[105] Schricker A.A., Lalani G.G., Krummen D.E., Rappel W.-J., Narayan S.M. (2014). Human atrial fibrillation initiates via organized rather than disorganized mechanisms. Circ. Arrhythm. Electrophysiol..

[106] Vidmar D., Narayan S.M., Rappel W.J. (2015). Phase synchrony reveals organization in human atrial fibrillation. Am. J. Physiol. Heart Circ. Physiol..

[107] Kowalewski C.A.B., Shenasa F., Rodrigo M., Clopton P., Meckler G., Alhusseini M.I., Swerdlow M.A., Joshi V., Hossainy S., Zaman J.A.B. (2018). Interaction of Localized Drivers and Disorganized Activation in Persistent Atrial Fibrillation: Reconciling Putative Mechanisms Using Multiple Mapping Techniques. Circ. Arrhythm. Electrophysiol..

[108] Takahashi Y., O’Neill M.D., Hocini M., Dubois R., Matsuo S., Knecht S., Mahapatra S., Lim K.-T., Jaïs P., Jonsson A. (2008). Characterization of electrograms associated with termination of chronic atrial fibrillation by catheter ablation. J. Am. Coll. Cardiol..

[109] Stiles M.K., Brooks A.G., Kuklik P., John B., Dimitri H., Lau D.H., Wilson L., Dhar S., Roberts-Thomson R.L., Mackenzie L. (2008). High-density mapping of atrial fibrillation in humans: Relationship between high-frequency activation and electrogram fractionation. J. Cardiovasc. Electrophysiol..

[110] Lazar S., Dixit S., Callans D.J., Lin D., Marchlinski F., Gerstenfeld E.P. (2006). Effect of pulmonary vein isolation on the left-to-right atrial dominant frequency gradient in human atrial fibrillation. Heart Rhythm..

[111] Krummen D.E., Peng K.A., Bullinga J.R., Narayan S.M. (2009). Centrifugal Gradients of Rate and Organization in Human Atrial Fibrillation. Pacing Clin. Electrophysiol..

[112] Coveney S., Corrado C., Roney C.H., O’Hare D., Williams S.E., O’Neill M.D., Niederer S.A., Clayton R.H., Oakley J.E., Wilkinson R.D. (2020). Gaussian process manifold interpolation for probabilistic atrial activation maps and uncertain conduction velocity. Philos. Trans. A Math. Phys. Eng. Sci..

[113] Sanders P., Nalliah C.J., Dubois R., Takahashi Y., Hocini M., Rotter M., Rostock T., Sacher F., Hsu L.-F., Jonsson A. (2006). Frequency mapping of the pulmonary veins in paroxysmal versus permanent atrial fibrillation. J. Cardiovasc. Electrophysiol..

[114] Petrutiu S., Sahakian A.V., Fisher W., Swiryn S. (2009). Manifestation of Left Atrial Events and Interatrial Frequency Gradients in the Surface Electrocardiogram During Atrial Fibrillation: Contributions from Posterior Leads. J. Cardiovasc. Electrophysiol..

[115] Ulphani J.S., Ng J., Aggarwal R., Cain J.H., Gordon D., Yang E., Morris A.R., Arora R., Goldberger J.J., Kadish A.H. (2007). Frequency gradients during two different forms of fibrillation in the canine atria. Heart Rhythm..

[116] Lim P.B., Malcolme-Lawes L.C., Stuber T., Kojodjojo P., Wright I.J., Francis D.P., Davies D.W., Peters N.S., Kanagaratnam P. (2011). Stimulation of the Intrinsic Cardiac Autonomic Nervous System Results in a Gradient of Fibrillatory Cycle Length Shortening Across the Atria During Atrial Fibrillation in Humans. J. Cardiovasc. Electrophysiol..

[117] Sarmast F., Kolli A., Zaitsev A., Parisian K., Dhamoon A.S., Guha P.K., Warren M., Anumonwo J.M., Taffet S.M., Berenfeld O. (2003). Cholinergic atrial fibrillation: *I*_K,ACh_ gradients determine unequal left/right atrial frequencies and rotor dynamics. Cardiovasc. Res..

[118] Mansour M., Mandapati R., Berenfeld O., Chen J., Samie F.H., Jalife J. (2001). Left-to-right gradient of atrial frequencies during acute atrial fibrillation in the isolated sheep heart. Circulation.

[119] Schuessler R.B., Kawamoto T., Hand D.E., Mitsuno M., Bromberg B.I., Cox J.L., Boineau J.P. (1993). Simultaneous epicardial and endocardial activation sequence mapping in the isolated canine right atrium. Circulation.

[120] Allessie M.A., de Groot N.M.S., Houben R.P.M., Schotten U., Boersma E., Smeets J.L., Crijns H.J. (2010). Electropathological Substrate of Long-standing Persistent Atrial Fibrillation in Patients with Structural Heart Disease: Longitudinal Dissociation. Circ. Arrhythm. Electrophysiol..

[121] Vigmond E., Pashaei A., Amraoui S., Cochet H., Hassaguerre M. (2016). Percolation as a mechanism to explain atrial fractionated electrograms and reentry in a fibrosis model based on imaging data. Heart Rhythm.

[122] Moe G.K., Rheinboldt W.C., Abildskov J.A. (1964). A computer model of atrial fibrillation. Am. Heart J..

[123] Allessie M.A., Lammers W., Smeets J., Bonke F.I.M., Hollen J. (1985). Experimental evaluation of Moe’s multiple wavelet hypothesis of atrial fibrillation. Cardiac Arrythmias 1985.

[124] Davidenko J.M., Pertsov A.M., Salomonsz R., Baxter W.P., Jalife J. (1992). Spatiotemporal irregularities of spiral wave activity in isolated ventricular muscle. J. Electrocardiol..

[125] Gray R.A., Pertsov A.M., Jalife J. (1998). Spatial and temporal organization during cardiac fibrillation. Nature.

[126] Pandit S.V., Jalife J. (2013). Rotors and the dynamics of cardiac fibrillation. Circ. Res..

[127] Hansen B.J., Briggs C., Moore B.T., Csepe T.A., Li N., Zhao J., Garikipati N.V., Janssen P.M., Mohler P.J., Hummel J.D. (2015). Human Atrial Fibrillation Drivers Seen Simultaneously by Focal Impulse and Rotor Mapping and High-resolution Optical Mapping. Circulation.

[128] Baykaner T., Rogers A.J., Meckler G.L., Zaman J., Navara R., Rodrigo M., Alhusseini M., Kowalewski C.A., Viswanathan M.N., Narayan S.M. (2018). Clinical Implications of Ablation of Drivers for Atrial Fibrillation: A Systematic Review and Meta-Analysis. Circ. Arrhythm. Electrophysiol..

[129] Honarbakhsh S., Schilling R.J., Providência R., Keating E., Chow A., Sporton S., Lowe M., Earley M.J., Lambiase P.D., Hunter R.J. (2018). Characterization of drivers maintaining atrial fibrillation: Correlation with markers of rapidity and organization on spectral analysis. Heart Rhythm.

[130] Hansen B.J., Zhao J., Li N., Zolotarev A., Zakharkin S., Wang Y., Atwal J., Kalyanasundaram A., Abudulwahed S.H., Helfrich K.M. (2018). Human Atrial Fibrillation Drivers Resolved With Integrated Functional and Structural Imaging to Benefit Clinical Mapping. JACC Clin. Electrophysiol..

[131] Anter E., Duytschaever M., Shen C., Strisciuglio T., Leshem E., Contreras-Valdes F.M., Waks J.W., Zimetbaum P.J., Kumar K., Spector P.S. (2018). Activation Mapping With Integration of Vector and Velocity Information Improves the Ability to Identify the Mechanism and Location of Complex Scar-Related Atrial Tachycardias. Circ. Arrhythm. Electrophysiol..

[132] Spector P.S., De Sa D.D.C., Tischler E.S., Thompson N.C., Habel N., Stinnett-Donnelly J., Benson B.E., Bielau P., Bates J.H. (2012). Ablation of multi-wavelet re-entry: General principles and in silico analyses. Europace.

[133] Baykaner T., Zografos T., Zaman J., Pantos I., Alhusseini M., Navara R., Krummen D.E., Narayan S.M., Katritsis D.G. (2017). Spatial relationship of organized rotational and focal sources in human atrial fibrillation to autonomic ganglionated plexi. Int. J. Cardiol..

[134] Ramirez F.D., Birnie D.H., Nair G.M., Szczotka A., Redpath C.J., Sadek M.M., Nery P.B. (2017). Efficacy and safety of driver-guided catheter ablation for atrial fibrillation: A systematic review and meta-analysis. J. Cardiovasc. Electrophysiol..

[135] Lin C.Y., Lin Y.-J., Narayan S.M., Baykaner T., Lo M.-T., Chung F.-P., Chen Y.-Y., Chang S.-L., Lo L.-W., Hu Y.-F. (2019). Comparison of phase mapping and electrogram-based driver mapping for catheter ablation in atrial fibrillation. Pacing Clin. Electrophysiol..

[136] Bellmann B., Lin T., Ruppersberg P., Zettwitz M., Guttmann S., Tscholl V., Nagel P., Roser M., Landmesser U., Rillig A. (2018). Identification of active atrial fibrillation sources and their discrimination from passive rotors using electrographical flow mapping. Clin. Res. Cardiol..

[137] Swerdlow M., Tamboli M., Ms M.A., Moosvi N., Rogers A., Leef G., Wang P., Rillig A., Brachmann J., Sauer W. (2019). Comparing phase and electrographic flow mapping for persistent atrial fibrillation. Pacing Clin. Electrophysiol..

[138] Seitz J., Bars C., Théodore G., Beurtheret S., Lellouche N., Bremondy M., Ferracci A., Faure J., Penaranda G., Yamazaki M. (2017). AF Ablation Guided by Spatiotemporal Electrogram Dispersion Without Pulmonary Vein Isolation: A Wholly Patient-Tailored Approach. J. Am. Coll. Cardiol..

[139] Qin M., Jiang W.-F., Wu S.-H., Xu K., Liu X. (2020). Electrogram dispersion-guided driver ablation adjunctive to high-quality pulmonary vein isolation in atrial fibrillation of varying durations. J. Cardiovasc. Electrophysiol..

[140] Honarbakhsh S., Hunter R.J., Ullah W., Keating E., Finlay M., Schilling R.J. (2019). Ablation in Persistent Atrial Fibrillation Using Stochastic Trajectory Analysis of Ranked Signals (STAR) Mapping Method. JACC Clin. Electrophysiol..

[141] Choudry S., Mansour M., Sundaram S., Nguyen D.T., Dukkipati S.R., Whang W., Kessman P., Reddy V.Y. (2020). RADAR: A Multicenter Food and Drug Administration Investigational Device Exemption Clinical Trial of Persistent Atrial Fibrillation. Circ. Arrhythm. Electrophysiol..

[142] Daoud E.G., Zeidan Z., Hummel J.D., Weiss R., Houmsse M., Augostini R., Kalbfleisch S.J. (2017). Identification of Repetitive Activation Patterns Using Novel Computational Analysis of Multielectrode Recordings During Atrial Fibrillation and Flutter in Humans. JACC Clin. Electrophysiol..

[143] Verma A., Sarkozy A., Skanes A., Duytschaever M., Bulava A., Urman R., Amos Y.A., De Potter T. (2018). Characterization and significance of localized sources identified by a novel automated algorithm during mapping of human persistent atrial fibrillation. J. Cardiovasc. Electrophysiol..

[144] Takahashi Y., Yamammoto T., Sekigawa M., Yamaguchi J., Shirai Y., Tao S., Hayashi T., Takigawa M., Goya M., Sasano T. (2020). Mapping After Pulmonary Vein Isolation in Persistent Atrial Fibrillation. Circ. Arrhythm. Electrophysiol..

[145] Grace A., Willems S., Meyer C., Verma A., Heck P., Zhu M., Shi X., Chou D., Dang L., Scharf C. (2019). High-resolution noncontact charge-density mapping of endocardial activation. JCI Insight.

[146] Shi R., Parikh P., Chen Z., Angel N., Norman M., Hussain W., Butcher C., Haldar S., Jones D.G., Riad O. (2019). Validation of Dipole Density Mapping during Atrial Fibrillation and Sinus Rhythm in Human Left Atrium. JACC Clin. Electrophysiol..

[147] Verma A., Narayan S.M. (2020). Getting in Contact With Atrial Fibrillation or Not. JACC Clin. Electrophysiol..

[148] Willems S., Verma A., Betts T., Murray S., Neuzil P., Ince H., Steven D., Sultan A., Heck P.M., Hall M.C. (2019). Targeting Nonpulmonary Vein Sources in Persistent Atrial Fibrillation Identified by Noncontact Charge Density Mapping. Circ. Arrhythm. Electrophysiol..

[149] Knecht S., Sohal M., Deisenhofer I., Albenque J.-P., Arentz T., Neumann T., Cauchemez B., Duytschaever M., Ramoul K., Verbeet T. (2017). Multicentre evaluation of non-invasive biatrial mapping for persistent atrial fibrillation ablation: The AFACART study. Europace.

[150] Haissaguerre M., Hocini M., Denis A., Shah A.J., Komatsu Y., Yamashita S., Daly M., Amraoui S., Zellerhoff S., Picat M.-Q. (2014). Driver domains in persistent atrial fibrillation. Circulation.

